# Chronic senolytic treatment alleviates established vasomotor dysfunction in aged or atherosclerotic mice

**DOI:** 10.1111/acel.12458

**Published:** 2016-08-05

**Authors:** Carolyn M. Roos, Bin Zhang, Allyson K. Palmer, Mikolaj B. Ogrodnik, Tamar Pirtskhalava, Nassir M. Thalji, Michael Hagler, Diana Jurk, Leslie A. Smith, Grace Casaclang‐Verzosa, Yi Zhu, Marissa J. Schafer, Tamara Tchkonia, James L. Kirkland, Jordan D. Miller

**Affiliations:** ^1^ Department of SurgeryMayo ClinicRochesterMNUSA; ^2^ Kogod Center on AgingMayo ClinicRochesterMNUSA; ^3^ Newcastle University Institute for AgingNewcastle UniversityNewcastle Upon TyneUK; ^4^ Department of Physiology & Biomedical EngineeringMayo ClinicRochesterMNUSA

**Keywords:** aging, atherosclerosis, endothelial function, calcification, fibrosis, senescence

## Abstract

While reports suggest a single dose of senolytics may improve vasomotor function, the structural and functional impact of long‐term senolytic treatment is unknown. To determine whether long‐term senolytic treatment improves vasomotor function, vascular stiffness, and intimal plaque size and composition in aged or hypercholesterolemic mice with established disease. Senolytic treatment (intermittent treatment with Dasatinib + Quercetin via oral gavage) resulted in significant reductions in senescent cell markers (TAF ^+^ cells) in the medial layer of aorta from aged and hypercholesterolemic mice, but not in intimal atherosclerotic plaques. While senolytic treatment significantly improved vasomotor function (isolated organ chamber baths) in both groups of mice, this was due to increases in nitric oxide bioavailability in aged mice and increases in sensitivity to NO donors in hypercholesterolemic mice. Genetic clearance of senescent cells in aged normocholesterolemic *INK‐ATTAC* mice phenocopied changes elicited by D+Q. Senolytics tended to reduce aortic calcification (alizarin red) and osteogenic signaling (qRT–PCR, immunohistochemistry) in aged mice, but both were significantly reduced by senolytic treatment in hypercholesterolemic mice. Intimal plaque fibrosis (picrosirius red) was not changed appreciably by chronic senolytic treatment. This is the first study to demonstrate that chronic clearance of senescent cells improves established vascular phenotypes associated with aging and chronic hypercholesterolemia, and may be a viable therapeutic intervention to reduce morbidity and mortality from cardiovascular diseases.

## Introduction

Risk factors for ischemic heart disease include hypercholesterolemia, arterial stiffness, chronic inflammation, hypertension, metabolic syndrome, and aging (Eckel *et al*., 2013). Importantly, these risk factors contribute to impaired endothelial function (Feletou & Vanhoutte, 2006), which can contribute to arterial remodeling and accelerate atherosclerotic plaque formation and expansion (Landmesser *et al*., 2004).

Recent work suggests senescent cell burden can be dramatically increased by chronological aging or in models of progeria (Lecka‐Czernik *et al*., 1997; Baker *et al*., 2004; Varela *et al*., 2005), high‐fat feeding (Shi *et al*., 2007), diabetes (Verzola *et al*., 2008), tobacco exposure (Nyunoya *et al*., 2006), or atherosclerosis (Wang & Bennett, 2012), and short‐term treatment with ‘senolytic’ drugs in chronologically aged or progeroid mice alleviates several aging‐related phenotypes (Zhu *et al*., 2015a,b). However, effects of long‐term senescent cell clearance on vascular reactivity and structure with aging or chronic hypercholesterolemia remain unknown.

## Can chronic senolytic treatment improve age‐related vascular pathology?

**Figure 1:**
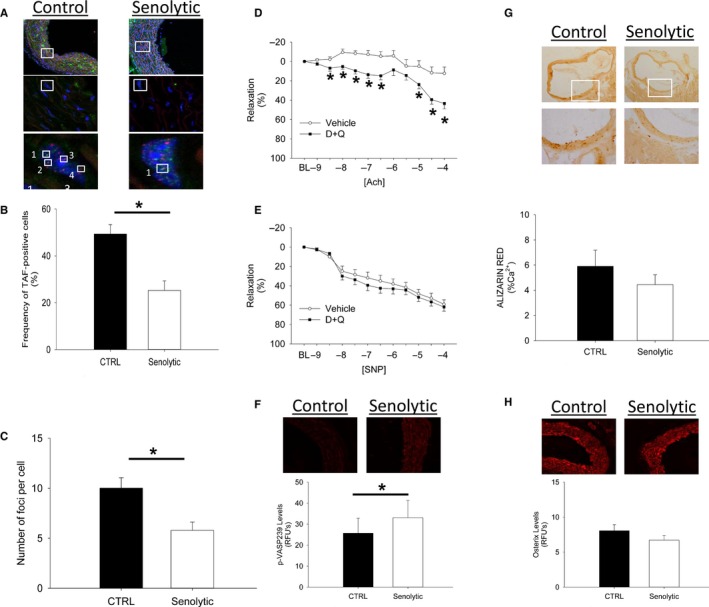
Effects of senolytic treatment on senescent cell burden, DNA damage, vasomotor function, nitric oxide signaling, calcification, and osteogenic signaling in chronologically aged mice. Chronic treatment with Dasatinib + Quercetin reduced senescent cell burden (A–B) and DNA damage (C) in aorta (Panel A pseudocolor legend for high magnification lower panels: DAPI/nuclei (blue), γH2A.X (green), telomeres (red). Low magnification micrographs in upper images are provided as an anatomic frame of reference/origin). White squares mark regions that are magnified in subsequent micrographs, and telomere‐associated foci are numbered in the bottom panels. Chronic senescent cell clearance improved vascular relaxation to acetylcholine (D) independently from changes in sodium nitroprusside (E) and significantly increased p‐VASP ^239^ levels (F). Chronic senolytic treatment with Dasatinib + Quercetin also tended to reduce vascular calcification (G), which was associated with modest reductions in osterix immunofluorescence (H). In all panels, asterisk denotes *p* < 0.05.

To determine whether senolytic treatment with dasatinib and quercetin (D+Q) reduces senescent cell burden and improves vascular function in aged mice, we maintained C57BL/6J mice on standard chow for 24 months, and then initiated vehicle or D+Q once monthly for 3 months (i.e., oral gavage with a dasatinib (5 mg kg^−1^)/quercetin (10 mg kg^−1^) cocktail once per month for months 24–27). TAF^+^ (telomere‐associated foci) nuclei were used as a marker of senescent cell burden (Hewitt *et al*., 2012). TAF^+^ nuclei were readily evident in subpopulations of cells in aorta (and localized in both endothelial and smooth muscle layers in the vessel wall) from vehicle‐treated mice (Fig. 1A–B and S1) and markedly reduced by D+Q (Fig. 1A–B and S1). Reductions in TAF^+^ cells were also associated with reductions in DNA damage (number of γ‐H2AX foci per cell) following D+Q (Fig. 1C).

In vehicle‐treated mice, carotid arteries showed minimal relaxation to acetylcholine assessed with an isolated organ chamber bath system (Miller *et al*., 2010), which was significantly improved in mice receiving intermittent D+Q for 3 months (Fig. 1D). In contrast to our previous study using a single dose of D+Q in aged mice, vascular relaxation to sodium nitroprusside was not changed by clearance of senescent cells in aged mice (Fig. 1E), and peak contractile responses increased following D+Q (Fig. S5, Supporting information). Similar senolytic efficacy and phenotypic changes were observed following genetic clearance of senescent cells in a subgroup of *INK‐ATTAC* mice [which allow for clearance of p16^ink4a^‐positive senescent cells via a caspase‐dependent mechanism (Baker *et al*., 2011)], thus confirming the effectiveness and phenotypic consequences of senescent cell clearance (Figs S2–S4). Collectively, these data suggest that acute senolytic treatment improves vasomotor function by increasing VSMC sensitivity to NO, whereas chronic, intermittent senolytic treatment improves vasomotor function by increasing NO bioavailability.

To probe mechanisms underlying improvements in vasomotor function, we assayed endothelial nitric oxide synthase phosphorylation levels (p‐eNOS^ser1177^, which are indicative of eNOS activation), but did not find significant differences between vehicle‐ and D+Q‐treated groups Fig. S6). Despite this finding, p‐VASP^239^ (Fig. 1F), a target of NO‐activated, cGMP‐dependent kinases (Sporbert *et al*., 1999), was significantly higher in D+Q‐treated aged mice. Although previous reports implicated reduced NOS cofactor production and increased Nox2‐derived free radicals in age‐associated endothelial dysfunction (Turgeon *et al*., 2012; Roos *et al*., 2013), we did not find significant changes in mRNA levels of NOS‐related or NADPH oxidase‐related enzymes following senolytic treatment (Figs S7–S9). Collectively, while our data suggest that senolytic treatment improves NO signaling in aged mice, precise molecular mechanisms underlying this phenomenon—including the potential contributions of other endothelium‐derived relaxing factors—have yet to be elucidated.

To determine whether senolytic treatment elicited structural changes in conduit arteries, we evaluated pressure–diameter relationships from carotid arteries in a pressurized organ chamber system (Matsumoto *et al*., 2007). No changes in carotid artery cross‐sectional compliance or distensibility were observed following pharmacological senescent cell clearance (Fig. S10). Medial vascular calcification in aorta, however, tended to be reduced by pharmacological cell clearance (Fig. 1G) and was paralleled by changes in protein levels of osterix (Fig. 1H). The modest effects of senolytic treatment on structural aspects of large arteries may be due to the period of time required to regress/reverse age‐related structural changes or the efficacy of senescent cell clearance (e.g., higher and/or more frequent dosing with senolytic agents) required to elicit regression of calcification or deleterious changes in vascular compliance in conduit vessels.

## Can chronic senolytic treatment improve hypercholesterolemia‐induced vascular pathology?

To determine whether senolytic treatment with D+Q reduces senescent cell burden and improves vascular function and structure in a model of atherosclerosis, ApoE^−/−^ mice were fed a Western diet (TD88137; Harlan Teklad) for 4 months to allow development of established atherosclerosis (Dansky *et al*., 1999). Vehicle or D+Q was then administered once weekly (i.e., oral gavage with a dasatinib (5 mg kg^−1^)/quercetin (10 mg kg^−1^) cocktail) for the next 2 months (i.e., months 4–6).

**Figure 2:**
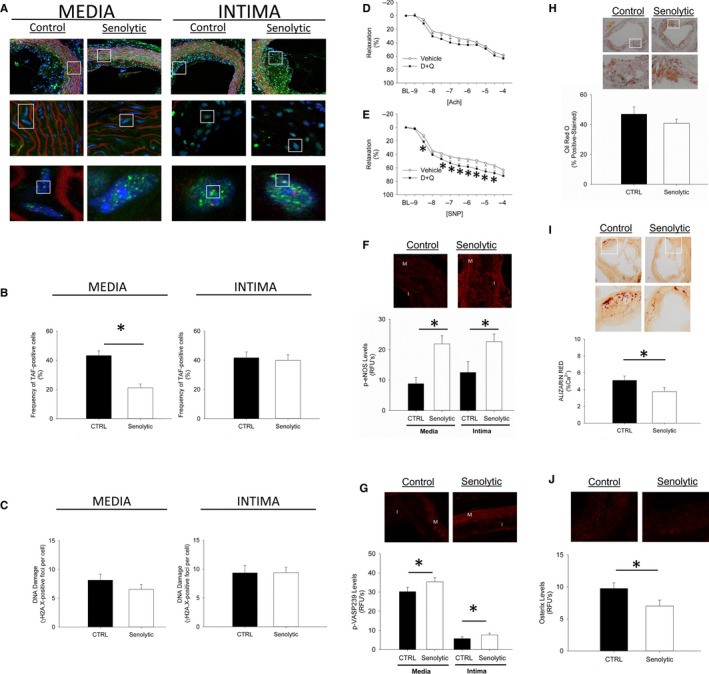
Effects of senolytic treatment on senescent cell burden, DNA damage, vasomotor function, and nitric oxide signaling in hypercholesterolemic mice. Chronic treatment with Dasatinib + Quercetin reduced senescent cell burden (A–B) and DNA damage (C) in the media of atherosclerotic plaques but not the intima (Panel A pseudocolor legend for high magnification lower panels: DAPI/nuclei (blue), γH2A.X (green), telomeres (red). Low magnification micrographs in upper images are provided as an anatomic frame of reference/origin). Chronic senescent cell clearance modestly improved vascular relaxation to acetylcholine (D) but markedly improved relaxation to sodium nitroprusside (E), and significantly increased levels of p‐eNOS ^ser1177^ (F) and p‐VASP ^239^ (G) in both the media and intima of atherosclerotic vessels. While chronic intermittent senolytic treatment with Dasatinib + Quercetin did not alter lipid composition of plaques (H), senolytic treatment did significantly reduce vascular calcification (I), which was associated with marked reductions in osterix immunofluorescence (J). In all panels, asterisk denotes *p* < 0.05.

Similarly to aged mice, TAF‐positive nuclei were abundant in the media and in intimal plaques of aorta from vehicle‐treated mice (Fig. 2A–B). Interestingly, senolytic treatment reduced senescent cell burden in medial segments of the vessel (Fig. 2A–B), but not in regions with established intimal atherosclerotic plaques (Fig. 2A–B). Indices of DNA damage paralleled reductions in senescent cell burden: reductions in DNA damage were evident only in medial segments of atherosclerotic vessels (Fig. 2C).

Unlike chronologically aged mice, D+Q did not improve responses to acetylcholine in isolated carotid arterial rings in an organ chamber bath system (Fig. 2D). We did, however, observe improved relaxation to sodium nitroprusside (Fig. 2E). Peak tension elicited by the thromboxane A2 agonist U46619 did not differ between treatment groups (Fig. S11).

Despite a lack of improvement in vasomotor responses to acetylcholine, we found that D+Q significantly increased levels of p‐eNOS^ser1177^ (Fig. 2F). Similarly to aging mice, we observed increases in p‐VASP^239^ in aorta (Fig. 2G), suggesting that basal NO signaling was improved in both medial and intimal regions of atherosclerotic vessels. Although we observed relatively large increase in p‐eNOS^ser1177^ levels, increases in p‐VASP^239^ levels remained small (particularly when compared to the relative reduction in p‐VASP^239^ levels in the intimal plaque region itself). When taken with the observation that senolytic treatment increased NADPH oxidase‐related protein in the intima (Figs S13–S14) in the absence of changes in enzymes related to NOS cofactor generation (Fig. S12), we conclude that changes in vasomotor function are likely the result of a complex interplay between nitric oxide bioavailability originating from the intima and oxidation state of sGC or downstream signal transduction molecules within the media (Landmesser *et al*., 2004; Feletou & Vanhoutte, 2006).

Regarding effects of senolytics on atherosclerotic plaque burden and composition in aorta, we found intimal plaque size was not affected in mice receiving D+Q for 2 months (Fig. S15), and analysis of Oil Red O‐stained sections suggested the overall lipid content of the plaque was also unaffected by senolytic treatment (Fig. 2H). Furthermore, expression of F4/80 in atherosclerotic vessels did not change appreciably (Fig. S16), suggesting negligible changes in overall macrophage burden. Among other possibilities, this could be related to intimal plaque penetrance by D+Q. Effects of newer senolytics, such as navitoclax (Zhu *et al*., 2015a,b), remain to be determined. We did, however, find that intimal plaque calcification was significantly reduced in D+Q‐ *vs*. vehicle‐treated mice (Fig. 2I). This was associated with reductions in protein levels of osterix (Fig. 2J). Intimal plaque fibrosis was not appreciably changed by senescent cell clearance (Fig. S17). This combination of findings is remarkable given the observations that calcified lesions are typically refractory to classic lipid‐lowering interventions and occurred in the absence of changes in plasma cholesterol levels (Fig. S18). These changes in plaque morphology are consistent, however, with previous reports demonstrating that senescent cells and cells overexpressing genes associated with progeroid syndromes have greater propensity to undergo osteogenic differentiation *in vitro* (Roos *et al*., 2013). Critically, unlike previous studies leveraging chronic treatment with compounds such as quercetin (e.g., administered in drinking water) (Hayek *et al*., 1997; Shen *et al*., 2013), our study shows that weekly, intermittent treatment is sufficient to elicit lasting molecular and functional consequences in the vasculature of hypercholesterolemic mice. Altogether, we believe these observations lay the groundwork for future studies aimed at understanding effects of senolytics on plaque composition and stability both in isolation and as a complementary therapy to lipid‐lowering strategies.

## Conclusions

Collectively, this study shows that chronic pharmacological clearance of senescent cells alleviates vasomotor dysfunction in naturally aging mice and mice with established atherosclerosis. Furthermore, senescent cell clearance reduces markers of osteogenesis in advanced intimal plaques, ultimately reducing intimal plaque calcification. Based on these findings, we conclude that senescent cell clearance may be an effective complementary therapy to classical risk factor management to reduce morbidity and mortality associated with age‐related cardiovascular diseases.

## Funding info

This work was supported by R01 HL111121 (JDM), R01 AG013925 (JLK), F30 AG046061 (AKP), Mayo Clinic Center for Regenerative Medicine, and the Connor Group, Noaber Foundation, and Ted Nash Foundation.

## Conflict of interest

JK, TT, YZ, TP, and AP have a financial interest related to this research. This research has been reviewed by the Mayo Clinic Conflict of Interest Review Board and is being conducted in compliance with Mayo Clinic Conflict of Interest policies.

## Supporting information


**Fig. S1.** Identification of TAF‐positive nuclei in subpopulations of cells in aorta from chronologically‐aged mice.
**Fig. S2.** Changes in whole tissue mRNA levels of a GFP reporter gene coupled to the senescence‐associated p16Ink4a promoter in chronologically aged mice.
**Fig. S3.** Changes in DNA damage following genetic or pharmacologic clearance of senescent cells in chronologically aged mice.
**Fig. S4.** Effects of genetic clearance of senescent cells in chronologically aged mice.
**Fig. S5.** Change in vasomotor responses to U46619 (a thromboxane A2 agonist) in carotid arteries from chronologically aged mice.
**Fig. S6.** Changes in levels of phosphorylated endothelial nitric oxide synthase (peNOSser1177) following senolytic treatment with D+Q. Note that chronic treatment with D+Q from ages 24 to 27 months did not alter levels of p‐eNOSser1177 compared to vehicle‐treated mice (CTRL).
**Fig. S7.** Changes in expression of nitric oxide synthase isoforms (A: eNOS, B: iNOS, C: nNOS) and enzymes related to nitric oxide synthase cofactor generation (D: GTPCH, E: DHFR) in aorta from chronologically aged mice treated with D+Q.
**Fig. S8.** Changes in mRNA levels of Nox2 in aorta from aged mice treated with D+Q.
**Fig. S9.** Change in mRNA and protein levels of Nox2 in aorta from chronologically aged mice treated with D+Q.
**Fig. S10.** Change in diameter (A), compliance (B), and distensibility (C) of carotid arteries from chronologically aged mice receiving senolytic treatment for 3 months.
**Fig. S11.** Change in vasomotor responses to U46619 (a thromboxane A2 agonist) in carotid arteries from hypercholesterolemic mice treated with D+Q for 2 months.
**Fig. S12.** Changes in expression of nitric oxide synthase isoforms (A: eNOS, B: iNOS, C: nNOS) and enzymes related to nitric oxide synthase cofactor generation (D: GTPCH, E: DHFR) in aorta from hypercholesterolemic mice following treatment with D+Q for 2 months.
**Fig. S13.** Changes in mRNA levels of Nox2 in aorta from hypercholesterolemic mice following treatment with D+Q for 2 months.
**Fig. S14.** Changes in protein levels of Nox2 in aorta from hypercholesterolemic mice following treatment with D+Q for 2 months.
**Fig. S15.** Changes in intimal plaque size in aorta from hypercholesterolemic mice following treatment with D+Q for 2 months.
**Fig. S16.** Changes in mRNA levels of F4/80 (a marker of macrophages) in aorta from hypercholesterolemic mice following weekly treatment with D+Q for 2 months.
**Fig. S17.** Changes in intimal plaque fibrosis in aorta from hypercholesterolemic mice following treatment with D+Q for 2 months.
**Fig. S18.** Changes in plasma cholesterol levels following weekly treatment with vehicle or D+Q for 2 months.

**Data S1.** Methods.

## References

[acel12458-bib-0001] Baker DJ , Jeganathan KB , Cameron JD , Thompson M , Juneja S , Kopecka A , Kumar R , Jenkins RB , de Groen PC , Roche P , van Deursen JM . (2004) BubR1 insufficiency causes early onset of aging‐associated phenotypes and infertility in mice. Nat. Genet. 36, 744–749. 15208629 10.1038/ng1382

[acel12458-bib-0002] Baker DJ , Wijshake T , Tchkonia T , LeBrasseur NK , Childs BG , van de Sluis B , Kirkland JL , van Deursen JM . (2011) Clearance of p16Ink4a‐positive senescent cells delays ageing‐associated disorders. Nature 479, 232–236. 22048312 10.1038/nature10600 PMC3468323

[acel12458-bib-0003] Dansky HM , Charlton SA , Sikes JL , Heath SC , Simantov R , Levin LF , Shu P , Moore KJ , Breslow JL , Smith JD . (1999) Genetic background determines the extent of atherosclerosis in ApoE‐deficient mice. Arterioscler. Thromb. Vasc. Biol. 19, 1960–1968. 10446078 10.1161/01.atv.19.8.1960

[acel12458-bib-0004] Eckel RH , Jakicic JM , Ard JD , de Jesus JM , Houston Miller N , Hubbard VS , Lee IM , Lichtenstein AH , Loria CM , Millen BE , Nonas CA , Sacks FM , Smith SC Jr , Svetkey LP , Wadden TA , Yanovski SZ , Kendall KA , Morgan LC , Trisolini MG , Velasco G , Wnek J , Anderson JL , Halperin JL , Albert NM , Bozkurt B , Brindis RG , Curtis LH , DeMets D , Hochman JS , Kovacs RJ , Ohman EM , Pressler SJ , Sellke FW , Shen WK , Tomaselli GF . (2013) AHA/ACC guideline on lifestyle management to reduce cardiovascular risk: a report of the American College of Cardiology/American Heart Association Task Force on Practice Guidelines. Circulation 129, S76–99. 24222015 10.1161/01.cir.0000437740.48606.d1

[acel12458-bib-0005] Feletou M , Vanhoutte PM (2006) Endothelial dysfunction: a multifaceted disorder (The Wiggers Award Lecture). Am. J. Physiol. Heart Circ. Physiol. 291, H985–1002. 16632549 10.1152/ajpheart.00292.2006

[acel12458-bib-0006] Hayek T , Fuhrman B , Vaya J , Rosenblat M , Belinky P , Coleman R , Elis A , Aviram M . (1997) Reduced progression of atherosclerosis in apolipoprotein E‐deficient mice following consumption of red wine, or its polyphenols quercetin or catechin, is associated with reduced susceptibility of LDL to oxidation and aggregation. Arterioscler. Thromb. Vasc. Biol. 17, 2744–2752. 9409251 10.1161/01.atv.17.11.2744

[acel12458-bib-0007] Hewitt G , Jurk D , Marques FD , Correia‐Melo C , Hardy T , Gackowska A , Anderson R , Taschuk M , Mann J , Passos JF . (2012) Telomeres are favoured targets of a persistent DNA damage response in ageing and stress‐induced senescence. Nat. Commun. 3, 708. 22426229 10.1038/ncomms1708 PMC3292717

[acel12458-bib-0008] Landmesser U , Hornig B , Drexler H (2004) Endothelial function: a critical determinant in atherosclerosis? Circulation 109, II27–33. 15173060 10.1161/01.CIR.0000129501.88485.1f

[acel12458-bib-0009] Lecka‐Czernik B , Moerman EJ , Shmookler RR , Lipschitz DA (1997) Cellular and molecular biomarkers indicate precocious in vitro senescence in fibroblasts from SAMP6 mice. Evidence supporting a murine model of premature senescence and osteopenia. J. Gerontol. A Biol. Sci. Med. Sci. 52, B331–336. 9402934 10.1093/gerona/52a.6.b331

[acel12458-bib-0010] Matsumoto T , Baker DJ , d'Uscio LV , Mozammel G , Katusic ZS , van Deursen JM . (2007) Aging‐associated vascular phenotype in mutant mice with low levels of BubR1. Stroke 38, 1050–1056. 17272762 10.1161/01.STR.0000257967.86132.01

[acel12458-bib-0011] Miller JD , Peotta VA , Chu Y , Weiss RM , Zimmerman K , Brooks RM , Heistad DD . (2010) MnSOD protects against COX1‐mediated endothelial dysfunction in chronic heart failure. Am. J. Physiol. Heart Circ. Physiol. 298, H1600–1607. 20304815 10.1152/ajpheart.01108.2009 PMC2867433

[acel12458-bib-0012] Nyunoya T , Monick MM , Klingelhutz A , Yarovinsky TO , Cagley JR , Hunninghake GW . (2006) Cigarette smoke induces cellular senescence. Am. J. Respir. Cell Mol. Biol. 35, 681–688. 16840774 10.1165/rcmb.2006-0169OC PMC2643295

[acel12458-bib-0013] Roos CM , Hagler M , Zhang B , Oehler EA , Arghami A , Miller JD . (2013) Transcriptional and phenotypic changes in aorta and aortic valve with aging and MnSOD deficiency in mice. Am. J. Physiol. Heart Circ. Physiol. 305, H1428–1439. 23997094 10.1152/ajpheart.00735.2012 PMC3840262

[acel12458-bib-0014] Shen Y , Ward NC , Hodgson JM , Puddey IB , Wang Y , Zhang D , Maghzal GJ , Stocker R , Croft KD . (2013) Dietary quercetin attenuates oxidant‐induced endothelial dysfunction and atherosclerosis in apolipoprotein E knockout mice fed a high‐fat diet: a critical role for heme oxygenase‐1. Free Radic. Biol. Med. 65, 908–915. 24017971 10.1016/j.freeradbiomed.2013.08.185

[acel12458-bib-0015] Shi Q , Hubbard GB , Kushwaha RS , Rainwater D , Thomas CA 3rd , Leland MM , Vandeberg JL , Wang XL . (2007) Endothelial senescence after high‐cholesterol, high‐fat diet challenge in baboons. Am. J. Physiol. Heart Circ. Physiol. 292, H2913–2920. 17277030 10.1152/ajpheart.01405.2006

[acel12458-bib-0016] Sporbert A , Mertsch K , Smolenski A , Haseloff RF , Schonfelder G , Paul M , Ruth P , Walter U , Blasig IE . (1999) Phosphorylation of vasodilator‐stimulated phosphoprotein: a consequence of nitric oxide‐ and cGMP‐mediated signal transduction in brain capillary endothelial cells and astrocytes. Brain Res. Mol. Brain Res. 67, 258–266. 10216224 10.1016/s0169-328x(99)00067-4

[acel12458-bib-0017] Turgeon J , Haddad P , Dussault S , Groleau J , Maingrette F , Perez G , Rivard A . (2012) Protection against vascular aging in Nox2‐deficient mice: impact on endothelial progenitor cells and reparative neovascularization. Atherosclerosis 223, 122–129. 22658259 10.1016/j.atherosclerosis.2012.05.003

[acel12458-bib-0018] Varela I , Cadinanos J , Pendas AM , Gutierrez‐Fernandez A , Folgueras AR , Sanchez LM , Zhou Z , Rodriguez FJ , Stewart CL , Vega JA , Tryggvason K , Freije JM , Lopez‐Otin C . (2005) Accelerated ageing in mice deficient in Zmpste24 protease is linked to p53 signalling activation. Nature 437, 564–568. 16079796 10.1038/nature04019

[acel12458-bib-0019] Verzola D , Gandolfo MT , Gaetani G , Ferraris A , Mangerini R , Ferrario F , Villaggio B , Gianiorio F , Tosetti F , Weiss U , Traverso P , Mji M , Deferrari G , Garibotto G . (2008) Accelerated senescence in the kidneys of patients with type 2 diabetic nephropathy. Am. J. Physiol. Renal. Physiol. 295, F1563–1573. 18768588 10.1152/ajprenal.90302.2008

[acel12458-bib-0020] Wang JC , Bennett M (2012) Aging and atherosclerosis: mechanisms, functional consequences, and potential therapeutics for cellular senescence. Circ. Res. 111, 245–259. 22773427 10.1161/CIRCRESAHA.111.261388

[acel12458-bib-0021] Zhu Y , Tchkonia T , Pirtskhalava T , Gower AC , Ding H , Giorgadze N , Palmer AK , Ikeno Y , Hubbard GB , Lenburg M , O'Hara SP , LaRusso NF , Miller JD , Roos CM , Verzosa GC , LeBrasseur NK , Wren JD , Farr JN , Khosla S , Stout MB , McGowan SJ , Fuhrmann‐Stroissnigg H , Gurkar AU , Zhao J , Colangelo D , Dorronsoro A , Ling YY , Barghouthy AS , Navarro DC , Sano T , Robbins PD , Niedernhofer LJ , Kirkland JL . (2015a) The Achilles’ heel of senescent cells: from transcriptome to senolytic drugs. Aging Cell 14, 644–658. 25754370 10.1111/acel.12344 PMC4531078

[acel12458-bib-0022] Zhu Y , Tchkonia T , Fuhrmann‐Stroissnigg H , Dai HM , Ling YY , Stout MB , Pirtskhalava T , Giorgadze N , Johnson KO , Giles CB , Wren JD , Niedernhofer LJ , Robbins PD , Kirkland JL . (2015b) Identification of a novel senolytic agent, navitoclax, targeting the Bcl‐2 family of anti‐apoptotic factors. Aging Cell 15, 428–435. 26711051 10.1111/acel.12445 PMC4854923

