# Supplementary material for: Chronic senolytic treatment alleviates established vasomotor dysfunction in aged or atherosclerotic mice
Source: Aging Cell. 2016 Aug 5;15(5):973–7. doi: 10.1111/acel.12458 (PMC5013022; doi:10.1111/acel.12458)
Supplement: Supplementary file 1 — Fig. S1. Identification of TAF‐positive nuclei in subpopulations of cells in aorta from chronologically‐aged mice. Fig. S2. Changes in whole tissue mRNA levels of a GFP reporter gene coupled to the senescence‐associated p16Ink4a promoter in chronologically aged mice. Fig. S3. Changes in DNA damage following genetic or pharmacologic clearance of senescent cells in chronologically aged mice. Fig. S4. Effects of genetic clearance of senescent cells in chronologically aged mice. Fig. S5. Change in vasomotor responses to U46619 (a thromboxane A2 agonist) in carotid arteries from chronologically aged mice. Fig. S6. Changes in levels of phosphorylated endothelial nitric oxide synthase (peNOSser1177) following senolytic treatment with D+Q. Note that chronic treatment with D+Q from ages 24 to 27 months did not alter levels of p‐eNOSser1177 compared to vehicle‐treated mice (CTRL). Fig. S7. Changes in expression of nitric oxide synthase isoforms (A: eNOS, B: iNOS, C: nNOS) and enzymes related to nitric oxide synthase cofactor generation (D: GTPCH, E: DHFR) in aorta from chronologically aged mice treated with D+Q. Fig. S8. Changes in mRNA levels of Nox2 in aorta from aged mice treated with D+Q. Fig. S9. Change in mRNA and protein levels of Nox2 in aorta from chronologically aged mice treated with D+Q. Fig. S10. Change in diameter (A), compliance (B), and distensibility (C) of carotid arteries from chronologically aged mice receiving senolytic treatment for 3 months. Fig. S11. Change in vasomotor responses to U46619 (a thromboxane A2 agonist) in carotid arteries from hypercholesterolemic mice treated with D+Q for 2 months. Fig. S12. Changes in expression of nitric oxide synthase isoforms (A: eNOS, B: iNOS, C: nNOS) and enzymes related to nitric oxide synthase cofactor generation (D: GTPCH, E: DHFR) in aorta from hypercholesterolemic mice following treatment with D+Q for 2 months. Fig. S13. Changes in mRNA levels of Nox2 in aorta from hypercholesterolemic mice following treatment [file ACEL-15-973-s001.pdf]

SUPPLEMENTAL DATA

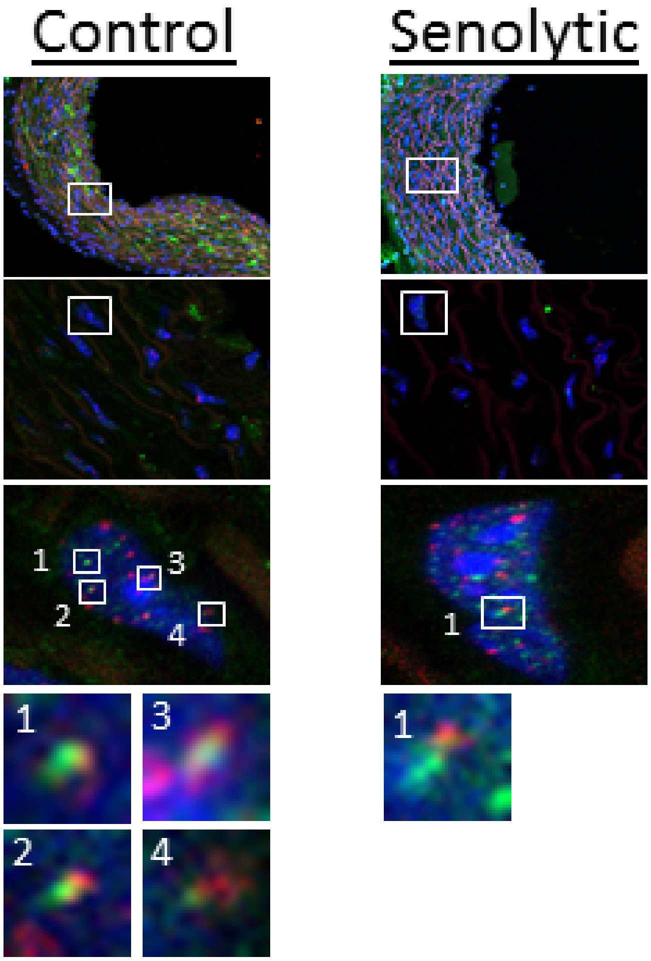

FIGURE S1. Identification of TAF-positive nuclei in subpopulations of cells in aorta from chronologically-aged mice. While upper panels depict the same images as shown in Figure 1, lower panels are high magnification representative images showing co-localization of telomeres with γ-H2AX immunostaining. Low magnification micrographs in upper images are provided as an anatomic frame of reference within the vessel wall and depict the origin of the lower micrographs. The pseudocolor legend for high magnification lower panels is as follows: DAPI/nuclei (blue), γH2A.X (green), and telomeres (red).

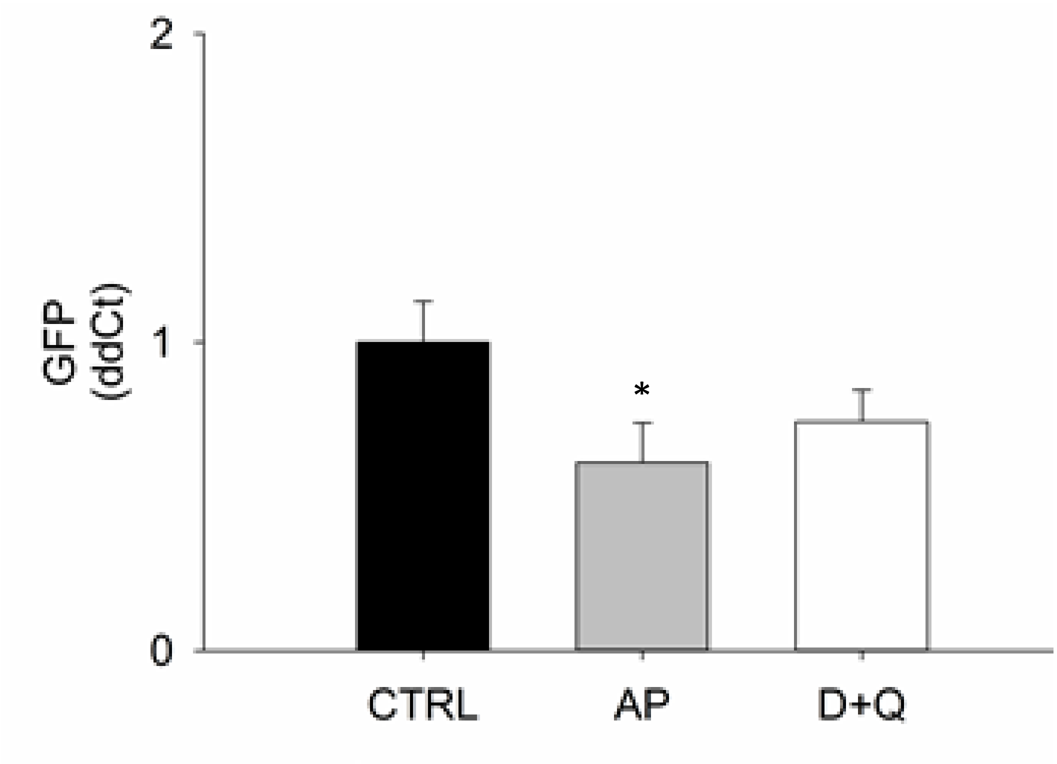

FIGURE S2. Changes in whole tissue mRNA levels of a GFP reporter gene coupled to the senescence-associated $p16^{Ink4a}$ promoter in chronologically aged mice. Consistent with a reduction in TAF^{+} cell number, we observed reductions in expression of the GFP reporter gene in following either genetic (AP20187 treatment in *INK-ATTAC* mice to clear $p16^{Ink4a}$-positive cells, * $p < 0.05$ vs CTRL) or pharmacologic (Dasatinib + Quercetin, $p = 0.07$ vs CTRL) clearance of senescent cells.

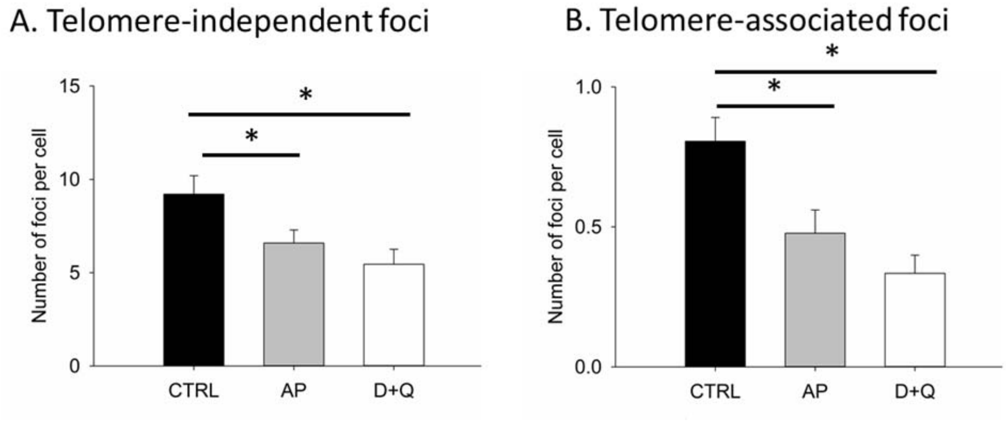

FIGURE S3. Changes in DNA damage following genetic or pharmacologic clearance of senescent cells in chronologically aged mice. The numbers of (**A**) telomere-independent γ-H2AX-positive nuclear foci and (**B**) telomere-associated γ-H2AX-positive nuclear foci were counted to assess DNA damage in aorta in control mice (i.e., vehicle-treated) and mice undergoing genetic or pharmacological clearance of senescent cells. Of note, these numbers (telomere-associated and telomere-independent) were summed together to obtain the "total" amount of DNA damage in Figure 1. Importantly, similar senolytic changes were observed in these subgroups of age- and sex-*INK-ATTAC* mice treated with AP20187 (AP 10 mg/kg) twice weekly from ages 24 to 27 months compared to D+Q treated mice.

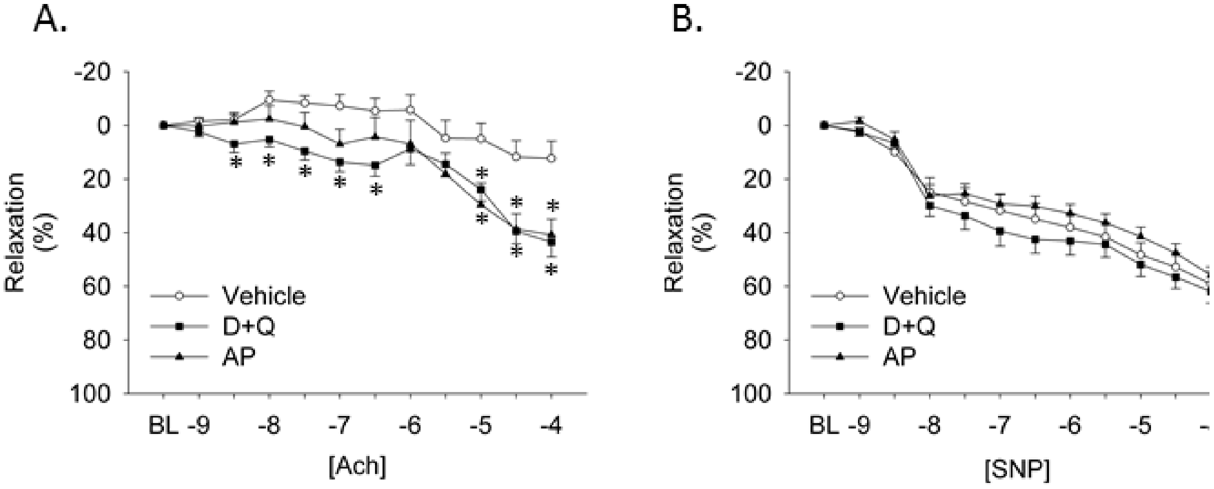

FIGURE S4. Effects of genetic clearance of senescent cells in chronologically aged mice. **A**: Chronic, intermittent activation of a "suicide gene" to clear senescent cells (using the previously described *INK-ATTAC* approach, Nature 479:232-236, 2011) significantly improved relaxation in response to acetylcholine compared to vehicle treated mice. Critically, similar phenotypic changes were observed in these subgroups of age- and sex-matched *INK-ATTAC* mice treated with AP20187 (AP, 10 mg/kg) twice weekly from ages 24 to 27 months compared to D+Q treated mice. **B**: Changes were not as pronounced in response to sodium nitroprusside (SNP).

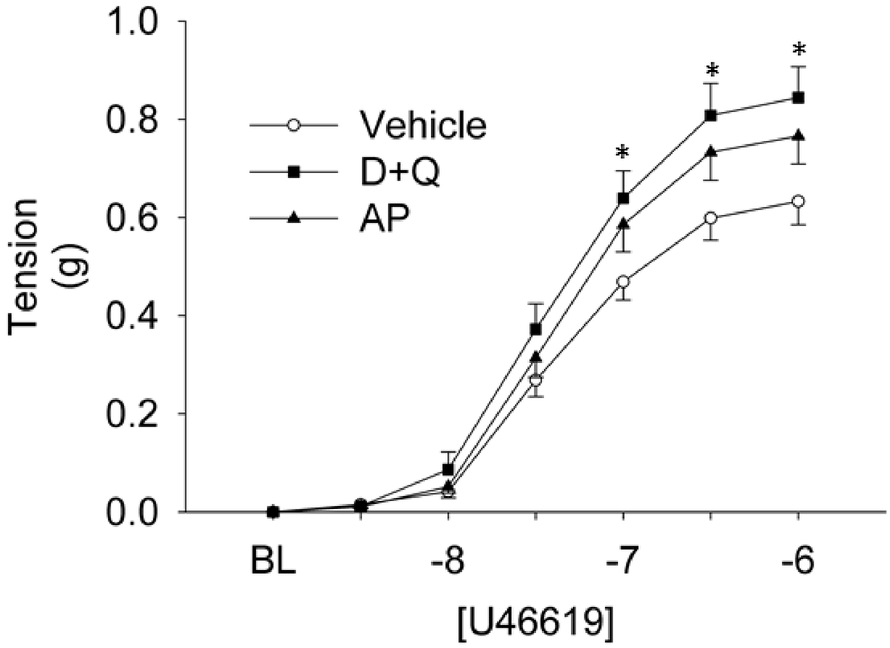

FIGURE S5. Change in vasomotor responses to U46619 (a thromboxane A2 agonist) in carotid arteries from chronologically aged mice. Carotid reactivity and sensitivity to U46619 reflects smooth muscle cell contractile function. Note that peak responses to U46619 are significantly increased following senolytic treatment, suggesting improved vascular smooth muscle function. As noted in previous figures, D+Q denotes C57BL/6J mice undergoing pharmacological senolytic treatment with a dasatinib/quercetin cocktail, and AP denotes background-matched INK-ATTAC mice undergoing genetic senolytic treatment.

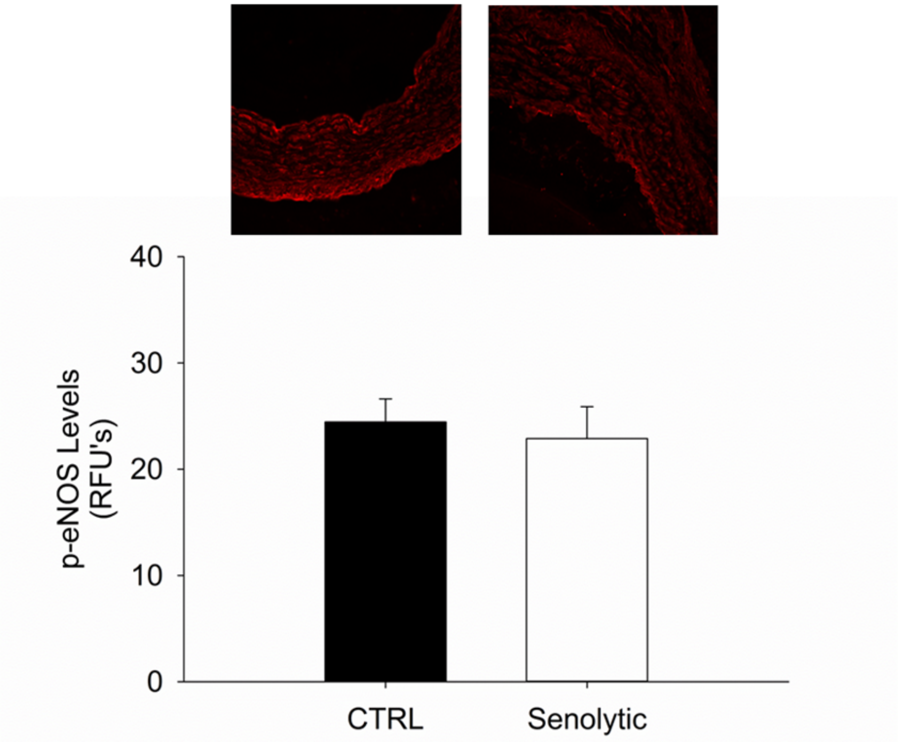

Figure S6. Changes in levels of phosphorylated endothelial nitric oxide synthase (p-eNOS^{ser1177}) following senolytic treatment with D+Q. Note that chronic treatment with D+Q from ages 24 to 27 months did not alter levels of p-eNOS^{ser1177} compared to vehicle-treated mice (CTRL).

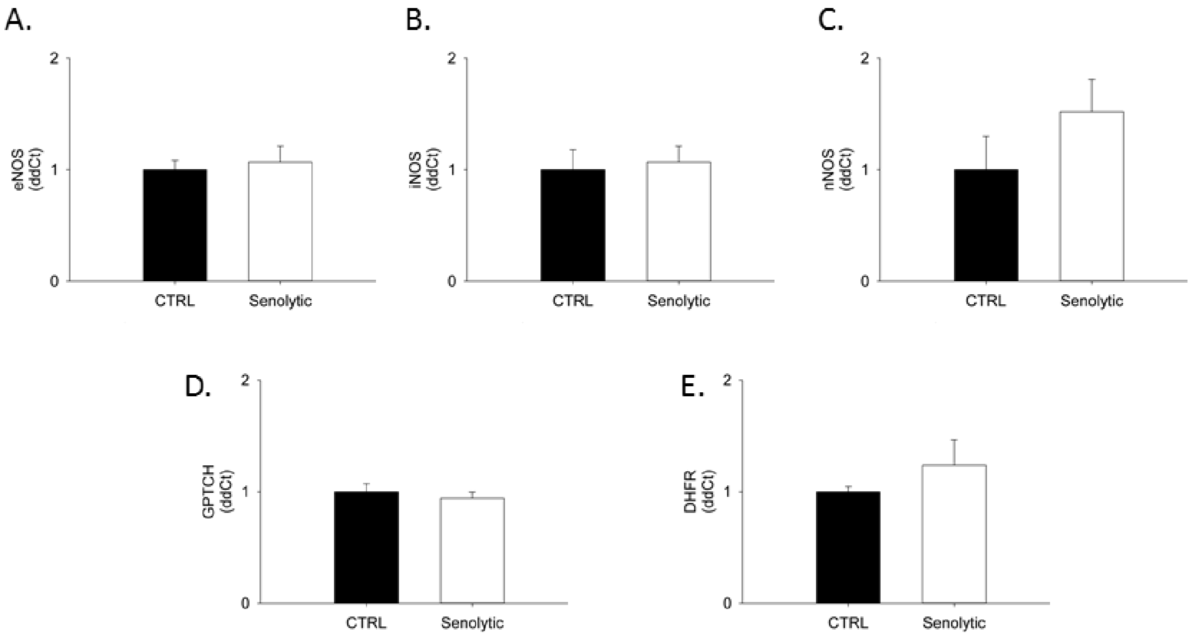

FIGURE S7. Changes in expression of nitric oxide synthase isoforms (**A**: eNOS, **B**: iNOS, **C**: nNOS) and enzymes related to nitric oxide synthase cofactor generation (**D**: GTPCH, **E**: DHFR) in aorta from chronologically aged mice treated with D+Q. Note that senolytic treatment with D+Q from ages 24 to 27 months did not significantly change mRNA levels of genes in either functional subgroup.

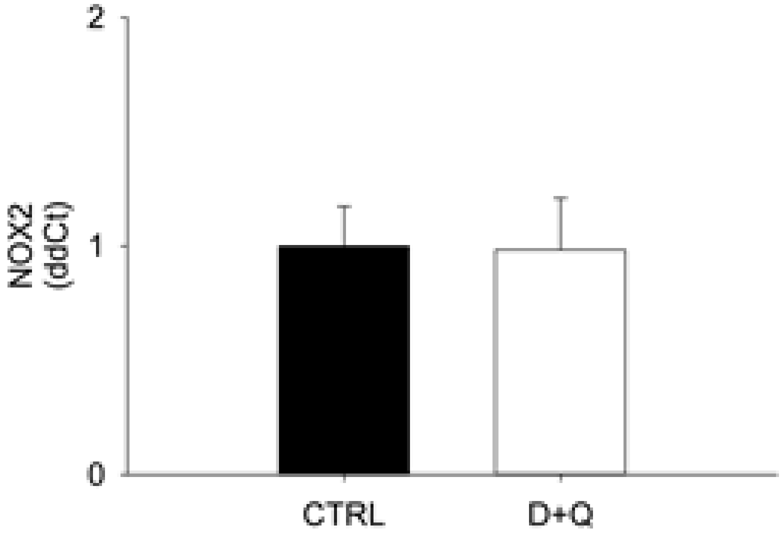

FIGURE S8. Changes in mRNA levels of Nox2 in aorta from aged mice treated with D+Q. Note that 3 months of senolytic treatment (from ages 24 to 27 months) did not significantly change mRNA levels of Nox2.

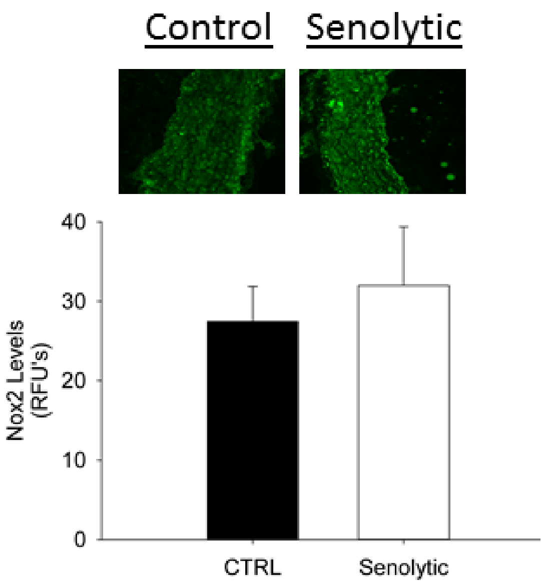

FIGURE S9. Change in mRNA and protein levels of Nox2 in aorta from chronologically aged mice treated with D+Q. Note that 3 months of senolytic treatment (from ages 24 to 27 months) did not significantly change mRNA levels of Nox2.

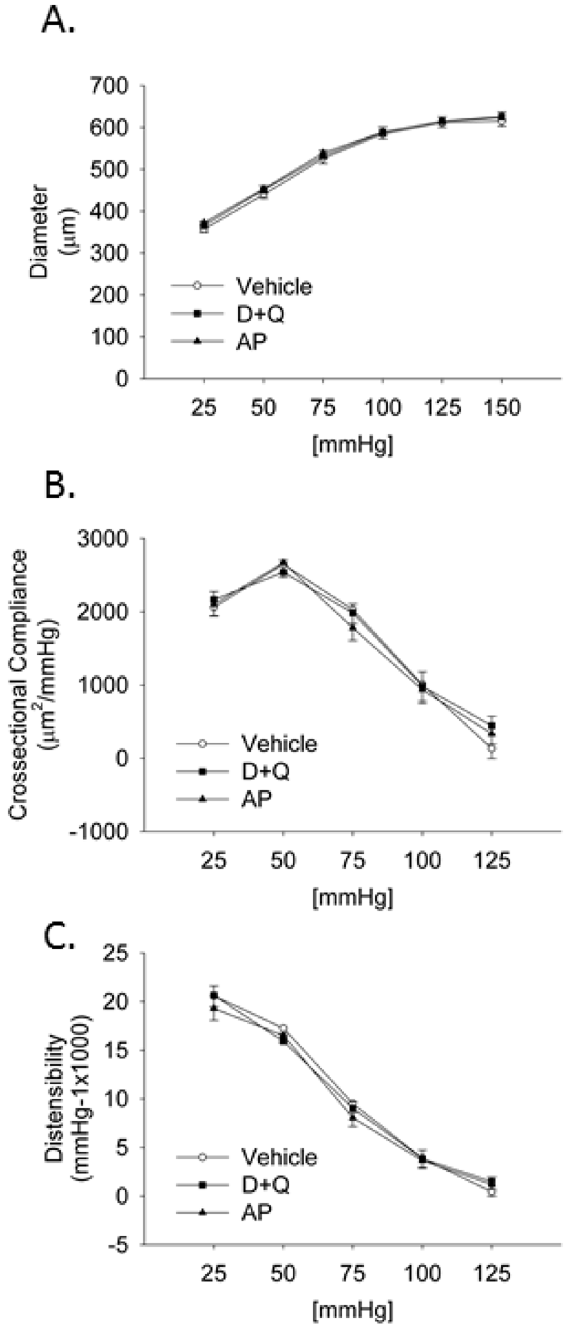

FIGURE S10. Change in diameter (**A**), compliance (**B**), and distensibility (**C**) of carotid arteries from chronologically aged mice receiving senolytic treatment for 3 months. Note that pharmacological (D+Q) or genetic (AP) clearance of senescent cells for 3 months does not significantly alter measures of vascular stiffness compared to vessels from vehicle-treated mice. Details of treatment with D+Q or AP20187can be found in the supplemental methods.

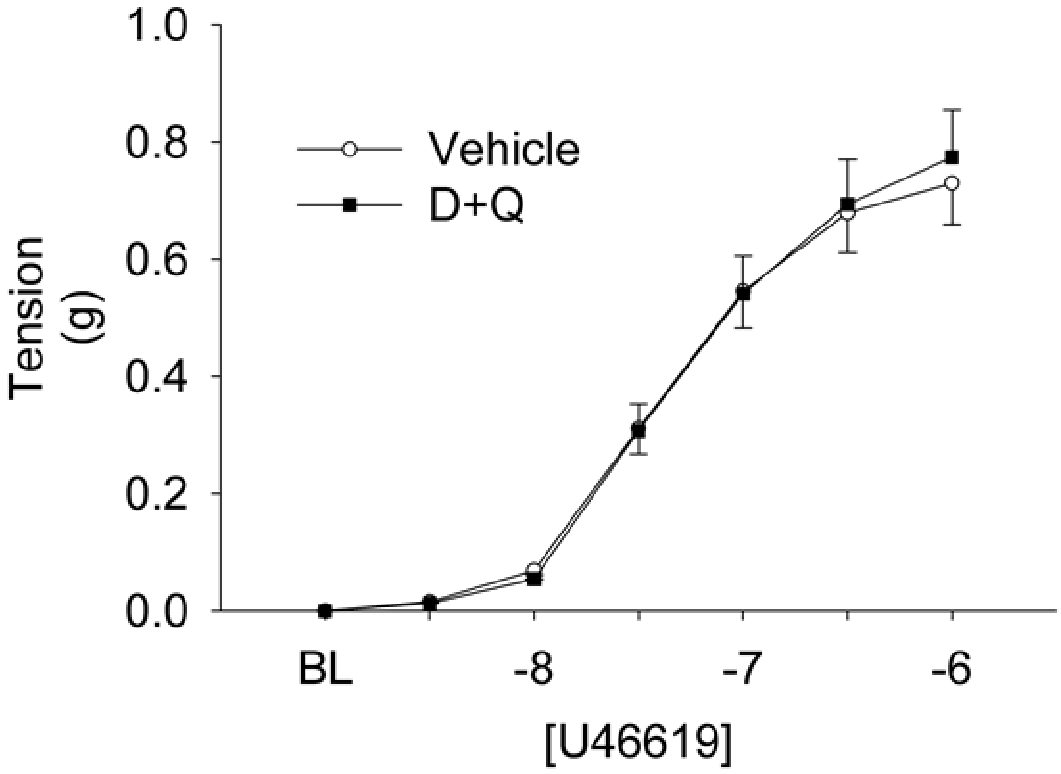

FIGURE S11. Change in vasomotor responses to U46619 (a thromboxane A2 agonist) in carotid arteries from hypercholesterolemic mice treated with D+Q for 2 months. Note that responses to U46619 are not significantly altered following pharmacological senolytic treatment with D+Q.

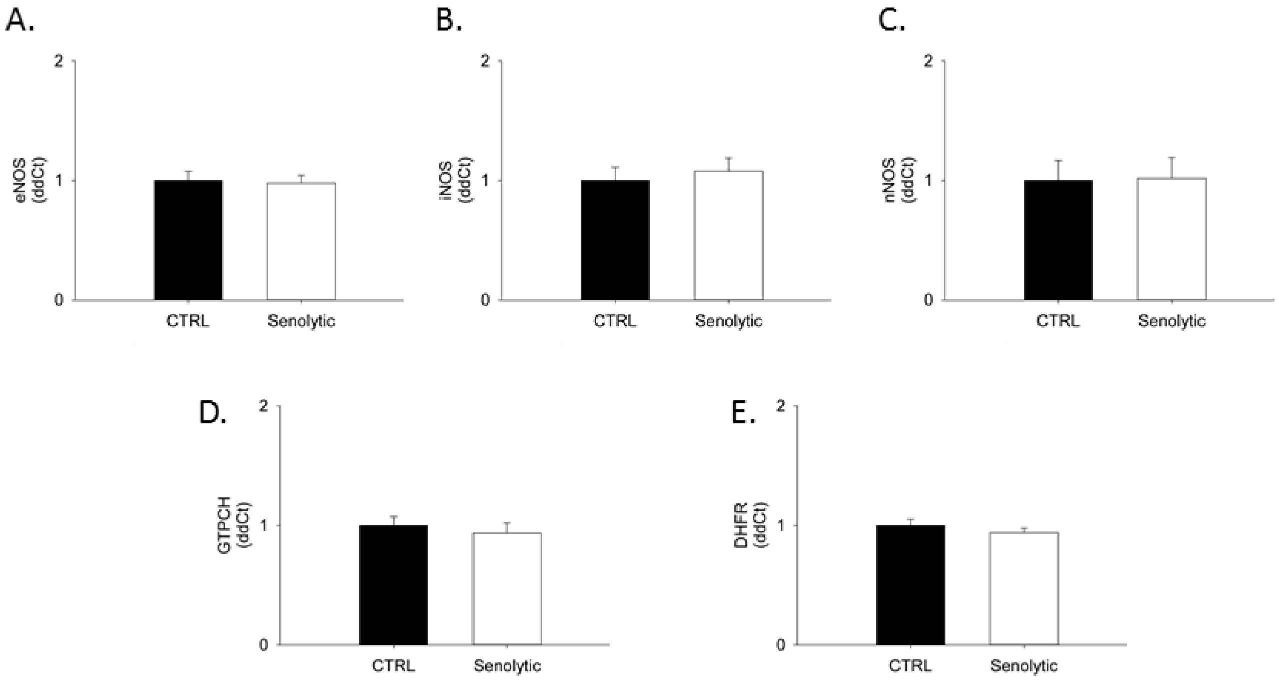

FIGURE S12. Changes in expression of nitric oxide synthase isoforms (**A**: eNOS, **B**: iNOS, **C**: nNOS) and enzymes related to nitric oxide synthase cofactor generation (**D**: GTPCH, **E**: DHFR) in aorta from hypercholesterolemic mice following treatment with D+Q for 2 months. Note that pharmacological senolytic treatment with D+Q did not significantly change mRNA levels of genes in either functional subgroup.

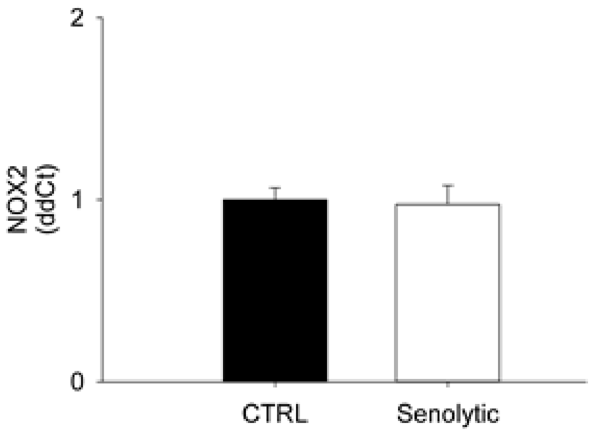

FIGURE S13. Changes in mRNA levels of Nox2 in aorta from hypercholesterolemic mice following treatment with D+Q for 2 months. Note that senolytic treatment did not significantly change mRNA levels of Nox2 following intermittent treatment with D+Q for 2 months.

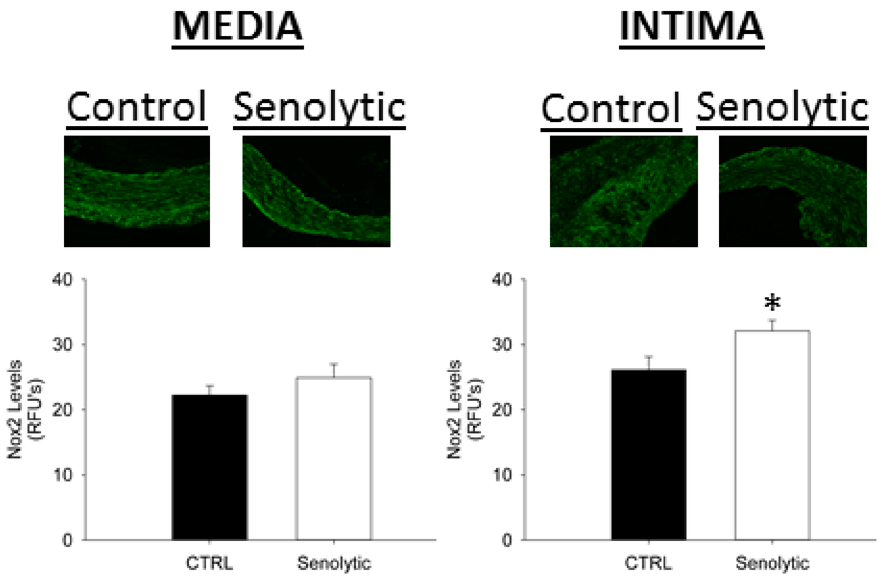

FIGURE S14. Changes in protein levels of Nox2 in aorta from hypercholesterolemic mice following treatment with D+Q for 2 months. Note that senolytic treatment with D+Q for 2 months significantly increased expression of Nox2 in regions of blood vessels containing intimal plaques.

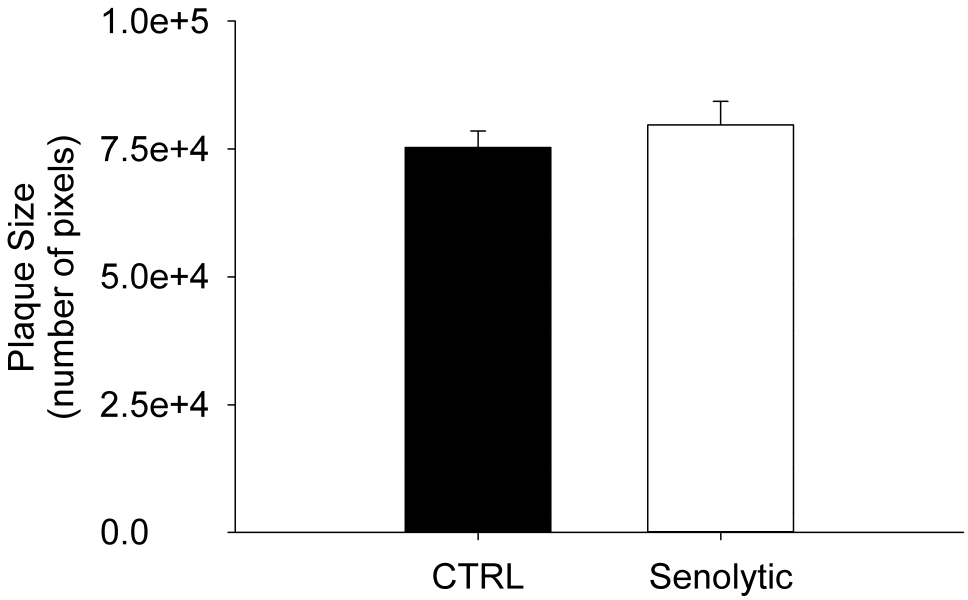

FIGURE S15. Changes in intimal plaque size in aorta from hypercholesterolemic mice following treatment with D+Q for 2 months. Note that pharmacological senolytic treatment did not alter the cross-sectional area of intimal plaques in mice treated with senolytics.

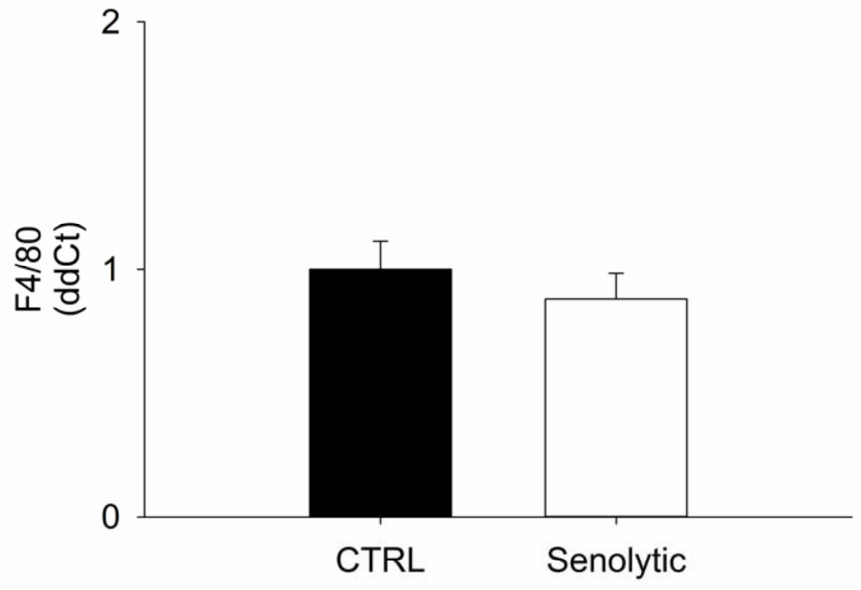

Figure S16. Changes in mRNA levels of F4/80 (a marker of macrophages) in aorta from hypercholesterolemic mice following weekly treatment with D+Q for 2 months. Note that pharmacological senolytic treatment did not significantly change mRNA levels of F4/80.

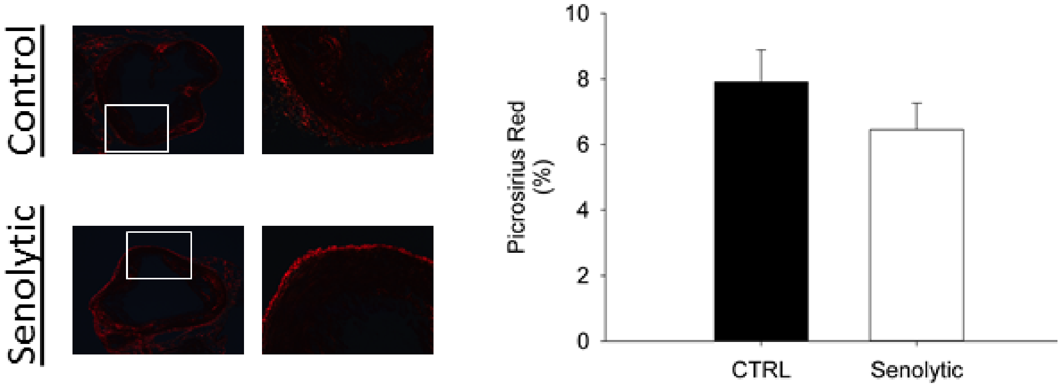

FIGURE S17. Changes in intimal plaque fibrosis in aorta from hypercholesterolemic mice following treatment with D+Q for 2 months. Note that chronic senolytic treatment did not significantly change measures of intimal plaque fibrosis in ApoE$^{-/-}$ mice.

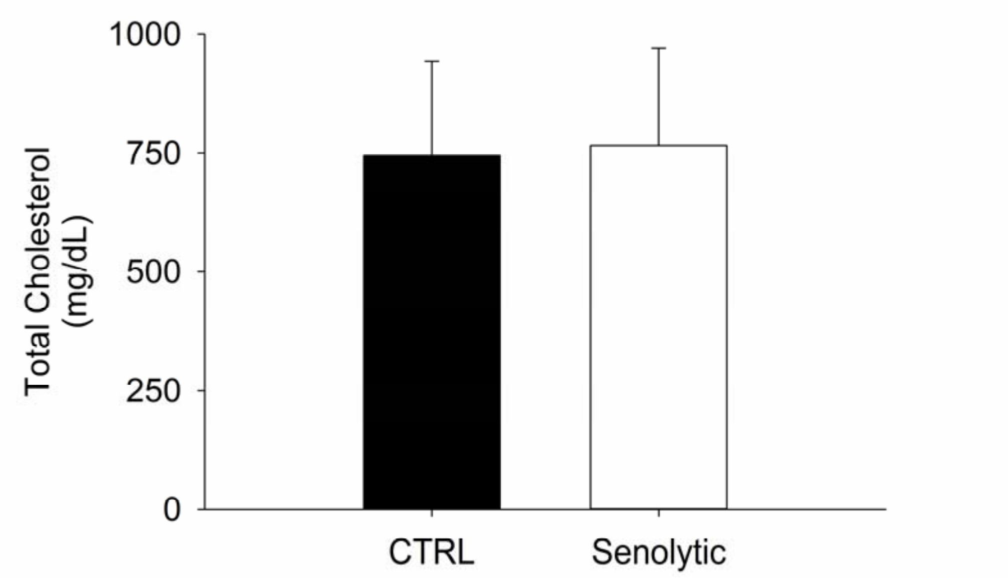

Figure S18. Changes in plasma cholesterol levels following weekly treatment with vehicle or D+Q for 2 months. Note that chronic, intermittent treatment with the pharmacological senolytic cocktail D+Q did not alter lipid levels.
