# Supplementary material for: Chronic senolytic treatment alleviates established vasomotor dysfunction in aged or atherosclerotic mice
Source: Aging Cell. 2016 Aug 5;15(5):973–7. doi: 10.1111/acel.12458 (PMC5013022; doi:10.1111/acel.12458)
Supplement: Supplementary file 2 — Data S1. Methods. [file ACEL-15-973-s002.docx]

**SUPPLEMENTAL METHODS**

*Chronological aging.* C57BL/6J mice were fed standard chow for 24 months. Vehicle or pharmacological senolytic treatment with D+Q was then administered once monthly for three months (*i.e*., months 24-27). A subgroup of age-, sex-, and background-matched *INK-ATTAC* mice were treated with AP20187 twice weekly from ages 24 to 27 months to confirm effectiveness and phenotypic consequences of senescent cell clearance. Mice were backcrossed to a C57BL/6J background by Dr. Jan van Deursen’s laboratory.

*Hypercholesterolemia.* Starting at 2 months of age, ApoE^-/-^ mice were fed a Western diet (TD88137; Harlan Teklad) for 4 months to allow development of established atherosclerosis[^1^](#_ENREF_1). Vehicle or senolytic treatment with D+Q was then administered once weekly for the next 2 months (*i.e*., months 4-6).

*Pharmacological senolytic treatment.* Mice were allocated to one of two oral gavage treatment groups: 1) dasatinib (5 mg/kg) plus quercetin (10 mg/kg) or 2) isovolumic vehicle. Treatment regimens for each group (aging or hypercholesterolemia) are denoted above.

*Genetic clearance of senescent cells.* Chronologically-aged *INK-ATTAC* mice were allocated to one of two intraperitoneal injection treatment groups: 1) AP20187 (10 mg/kg) or 2) isovolumic vehicle. p16Ink4a positive senescent cells can be selectively eliminated from INK-ATTAC mice. *INK-ATTAC* mice express a transgene comprising a senescence-activated p16^Ink4a^ promoter sequence that drives a drug-activatable "suicide" gene product, ATTAC (apoptosis through targeted activation of caspase). ATTAC encodes a mutated FK506 binding protein (FKBP)/ caspase-8 fusion protein. A drug without effect on normal cells, AP20187, cross-links the mutated FKBP in the membrane-bound myristoylated ATTAC fusion protein. This dimerizes and activates ATTAC caspase-8 moieties, resulting in apoptosis, even in non-dividing cells such as senescent cells.


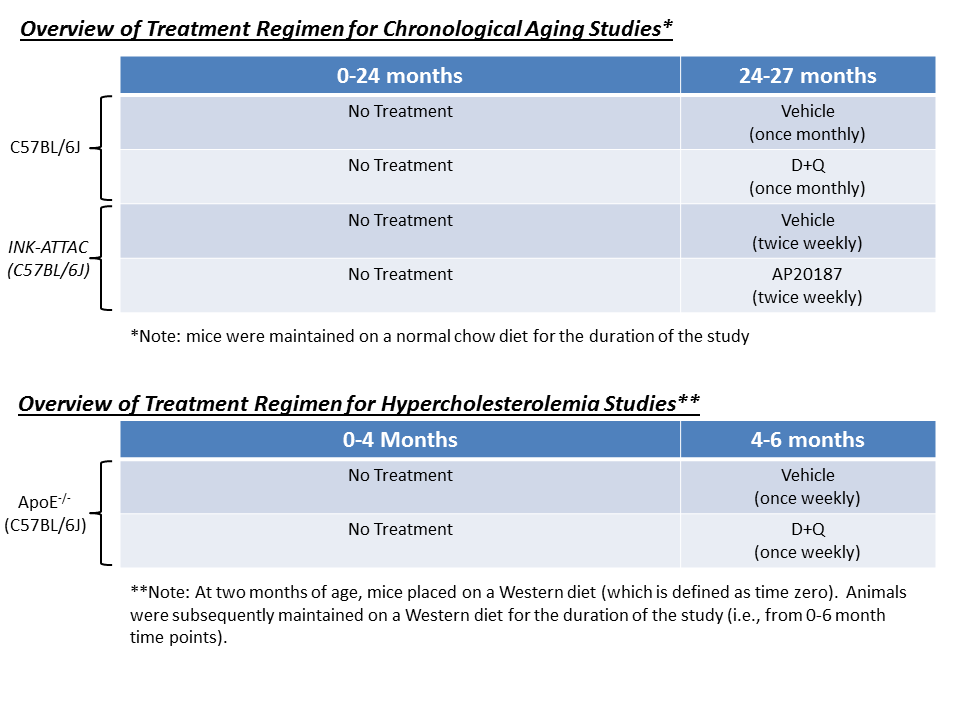


*Detection of senescent cells*. Senescent cell abundance is low, even in tissues of old animals [^2^](#_ENREF_2). This makes it difficult to detect declines in mRNA’s, such as p16, which are also present in many normal cell types that are also present in whole tissue. Senescence-associated β-galactosidase (SA β-Gal) can be used to detect senescent cells, but has limited sensitivity and selectivity[^3^](#_ENREF_3)^,^ [^4^](#_ENREF_4). For example, SA β-Gal is frequently expressed by activated macrophages[^5^](#_ENREF_5), suggesting it could be unreliable for distinguishing the macrophages from senescent cells in atherosclerotic plaques. Furthermore, background SA β-Gal is high in muscle, further complicating use of this assay for detecting senescent cells in the media of arteries. Therefore, we used 3 other assays to detect senescent cells: 1) telomere-associated foci (TAF’s), a recently developed method for detecting DNA damage foci within telomeric DNA that appears to be more highly sensitive and specific for detecting senescent cells than other methods [^6^](#_ENREF_6); 2) GFP mRNA in *INK-ATTAC* mice, in which a senescence-activated p16^Ink4a^ promoter fragment drives GFP; and 3) nuclear γ-H2AX foci, which reflect DNA damage and that are more frequent in senescent (>3 foci/nucleus) than non-senescent cells[^7^](#_ENREF_7).

*γ-H2AX and Telomere-Associated Foci*. Sections of OCT-embedded tissue were mounted on glass slides and processed for γH2A.X and telomere regions as previously described[^6^](#_ENREF_6)^,^ [^8^](#_ENREF_8). γH2A.X antibody was purchased from Cell Signaling. Biotinylated secondary and Fluorescein Avidin DCS antibodies were purchased from Vector Laboratories. Following γH2A.X immunofluorescence, telomere immunoFISH was performed using a Cy-3-labelled telomere-specific (CCCTAA) peptide nucleic acid probe (Panagene, Daejeon, KR). Counter-staining was done with DAPI and images were taken and in-depth Z stacking was used (a minimum of 40 optical slices with ×63 objective).

*Gene expression.* Thoracic aorta was removed *en bloc*, and perivascular adipose tissue and adventitial tissue was removed. The aortic arch was snap frozen in liquid nitrogen, pulverized, and resuspended in lysis buffer. RNA was isolated using spin columns and chloroform extractions (Invitrogen). After conversion to cDNA (VILO reverse transcriptase, Invitrogen), quantitative real-time PCR was performed on a StepOne Plus RT-PCR machine (Applied Biosystems).

*Vasomotor Function.* Rings from carotid arteries were used for vasomotor function studies. In brief, excess adventitial tissue and perivascular fat were removed, and sections of approximately 2-3 mm in length were mounted on stainless-steel hooks. The vessels were maintained in an organ bath chamber containing oxygenated (95% O_2_/5% CO_2_) Krebs solution at 37°C, and allowed to equilibrate for 1 hour prior to evaluation of vasomotor function. Vessels were rinsed for a minimum of 30 minutes between vasomotor function curves. Responses to acetylcholine, nitroprusside, and U46619 were evaluated.

*Vascular stiffness.* Rings from carotid arteries were used for vascular stiffness studies. In brief, excess adventitial tissue and perivascular fat were removed, and sections of approximately 2-3 mm in length were sutured onto microcannulae and placed in a vessel chamber (Living Systems Instrumentation) containing Ca^2+^-free, EDTA chelated, and oxygenated (95% O_2_/5% CO_2_) Krebs solution at 37°C, and allowed to equilibrate for 1 hour prior to evaluation of vascular stiffness. In brief, diameter changes were measured during stepwise increments in intraluminal pressure from 25 to 150 mm Hg. Passive diameter was defined as the inner diameter of carotid artery. Cross-sectional compliance was calculated as the change in luminal cross-sectional area for a given change in intravascular pressure (*i.e.,* ΔCSA/ΔP). Distensibility was defined as the compliance value normalized for the luminal cross-sectional area prior to a pressure increment.

*Measurement of changes in vascular calcification, lipid content, and fibrous content of intimal plaques.* Calcium levels were measured in cryosections of aorta using Alizarin Red staining followed by semi-quantitative analysis using Adobe Photoshop, as described previously[^9^](#_ENREF_9)^,^ [^10^](#_ENREF_10). Lipid content of intimal plaques was measured in cryosections of aorta using oil red O staining followed by semi-quantitative analysis using Adobe Photoshop, as described previously[^9^](#_ENREF_9)^,^ [^10^](#_ENREF_10). Collagen levels were measured in cryosections of aorta using Picrosirius Red staining imaged using polarized light microscopy. Semi-quantitative analysis of fibrous content was performed by evaluating the percentage of intimal plaques displaying collagen birefringence across red, orange, green, and yellow color spectra.

*Immunohistochemistry*. Protein levels of p-eNOS^ser1177^, p-VASP^239^, Nox2, and osterix were measured in cryosections of aorta using established fluorescent immunohistochemical techniques. Alexa Fluor 647 secondary antibodies were used to avoid changes in tissue autofluorescence secondary to alterations in calcium levels or collagen content.

**REFERENCES**

1. Dansky HM, Charlton SA, Sikes JL, Heath SC, Simantov R, Levin LF, Shu P, Moore KJ, Breslow JL, Smith JD. Genetic background determines the extent of atherosclerosis in apoe-deficient mice. *Arterioscler Thromb Vasc Biol*. 1999;19:1960-1968

2. Jeyapalan JC, Ferreira M, Sedivy JM, Herbig U. Accumulation of senescent cells in mitotic tissue of aging primates. *Mech Ageing Dev*. 2007;128:36-44

3. Dimri GP, Lee X, Basile G, Acosta M, Scott G, Roskelley C, Medrano EE, Linskens M, Rubelj I, Pereira-Smith O, et al. A biomarker that identifies senescent human cells in culture and in aging skin in vivo. *Proc Natl Acad Sci U S A*. 1995;92:9363-9367

4. Yang NC, Hu ML. The limitations and validities of senescence associated-beta-galactosidase activity as an aging marker for human foreskin fibroblast hs68 cells. *Exp Gerontol*. 2005;40:813-819

5. Kurz DJ, Decary S, Hong Y, Erusalimsky JD. Senescence-associated (beta)-galactosidase reflects an increase in lysosomal mass during replicative ageing of human endothelial cells. *J Cell Sci*. 2000;113 ( Pt 20):3613-3622

6. Jurk D, Wilson C, Passos JF, Oakley F, Correia-Melo C, Greaves L, Saretzki G, Fox C, Lawless C, Anderson R, Hewitt G, Pender SL, Fullard N, Nelson G, Mann J, van de Sluis B, Mann DA, von Zglinicki T. Chronic inflammation induces telomere dysfunction and accelerates ageing in mice. *Nat Commun*. 2014;2:4172

7. d'Adda di Fagagna F. Living on a break: Cellular senescence as a DNA-damage response. *Nat Rev Cancer*. 2008;8:512-522

8. Hewitt G, Jurk D, Marques FD, Correia-Melo C, Hardy T, Gackowska A, Anderson R, Taschuk M, Mann J, Passos JF. Telomeres are favoured targets of a persistent DNA damage response in ageing and stress-induced senescence. *Nat Commun*. 2012;3:708

9. Miller JD, Weiss RM, Serrano KM, Brooks RM, 2nd, Berry CJ, Zimmerman K, Young SG, Heistad DD. Lowering plasma cholesterol levels halts progression of aortic valve disease in mice. *Circulation*. 2009;119:2693-2701

10. Miller JD, Weiss RM, Serrano KM, Castaneda LE, Brooks RM, Zimmerman K, Heistad DD. Evidence for active regulation of pro-osteogenic signaling in advanced aortic valve disease. *Arterioscler Thromb Vasc Biol*. 2010;30:2482-2486
